# Ultrasound for the Detection of Gallbladder Malignancy in Primary Sclerosing Cholangitis

**DOI:** 10.1111/liv.70312

**Published:** 2025-08-26

**Authors:** Johannes Altenmüller, Christiane Wiegard, Marcial Sebode, Ansgar W. Lohse, Christina Villard, Stergios Kechagias, Emma Nilsson, Fredrik Rorsman, Hanns‐Ulrich Marschall, Kalle Jokelainen, Annika Bergquist, Martti Färkkilä, Christoph Schramm

**Affiliations:** ^1^ Ist Department of Medicine University Medical Center Hamburg‐Eppendorf Hamburg Germany; ^2^ Ist Derparmtent of Medicine Westküstenklinikum Heide Heide Germany; ^3^ European Reference Network on Hepatological Diseases (ERN RARE‐LIVER) Hamburg Germany; ^4^ Hamburg Center for Translational Immunology University Medical Center Hamburg‐Eppendorf Hamburg Germany; ^5^ Department of Transplantation Surgery Karolinska University Hospital, Department of Medicine Huddinge, Karolinska Institutet Stockholm Sweden; ^6^ Unit of Internal Medicine, Department of Health, Medicine and Caring Sciences Linköping University Linköping Sweden; ^7^ Department of Clinical Sciences, Lund University, Gastroenterology Clinic Skåne University Hospital Lund Sweden; ^8^ Department of Gastroenterology and Hepatology University Hospital Uppsala Sweden; ^9^ Institute of Medicine, Sahlgrenska Academy, University of Gothenburg, and Department of Medicine Sahlgrenska University Hospital Gothenburg Sweden; ^10^ Clinic of Gastroenterology Helsinki University and Helsinki University Hospital Helsinki Finland; ^11^ Department of Medicine Huddinge, Unit of Gastroenterology and Rheumatology Karolinska Institutet, Karolinska University Hospital Stockholm Sweden; ^12^ Martin Zeitz Centre for Rare Diseases University Medical Center Hamburg‐Eppendorf Hamburg Germany

**Keywords:** cholecystectomy, imaging, malignancy, PSC

## Abstract

**Background and Aims:**

In primary sclerosing cholangitis (PSC), the risk for gallbladder malignancy is increased. Surveillance imaging is used for early diagnosis. The study aims to assess the reliability of ultrasound and magnetic resonance imaging (MRI) for the detection of gallbladder polyps in people with PSC and to define a polyp size as a cut‐off at which cholecystectomy is indicated due to the high probability of a malignant finding.

**Methods:**

In this retrospective European multicentre study, we included 51 people with PSC who had cholecystectomy for gallbladder polyps detected on imaging using ultrasound and/or MRI within 6 months prior to cholecystectomy and a histology report available. As a control group, we included 102 people with PSC with other indications for cholecystectomy. Malignancy was defined as high‐grade dysplasia or carcinoma on histology.

**Results:**

Including all 153 patients, ultrasound was significantly more sensitive than MRI in detecting gallbladder polyps (*p* < 0.001). MRI missed 3 of the 8 malignant polyps. Malignant polyps (*n* = 8, median size = 12.5 mm) were significantly larger than non‐malignant polyps (*n* = 26, median size = 6 mm) on ultrasound (*p* < 0.001). Ultrasound detected malignant polyps reliably (AUC = 0.91, *p* < 0.001) with an optimal cut‐off of 8 mm. This cut‐off was defined in the Hamburg cohort and validated in a multicentre validation cohort with an AUC of 0.92 (*p* = 0.02).

**Conclusions:**

Ultrasound is more sensitive for the detection of gallbladder polyps than MRI in people with PSC. The best cut‐off to differentiate between benign and malignant polyps was 8 mm. Ultrasound (gallbladder) and MRI (bile ducts) may thus be complementary methods for hepatobiliary malignancy surveillance in people with PSC.

**Summary**

- Primary sclerosing Cholangitis is an inflammatory disease of the bile ducts that is associated with an increased risk of bile duct and gallbladder cancer, which can develop from gallbladder polyps.
- Imaging examinations using ultrasound and magnetic resonance imaging are used for the early detection of these lesions.
- We here show that gallbladder polyps larger than 8 mm have a high likelihood to be malignant, indicating surgical removal of the gallbladder.

**Abbreviations**
AUC: area under the curve
CEUS: contrast enhanced ultrasound
MELD: model for end‐stage liver disease
MRI: magnetic resonance imaging
PSC: primary sclerosing cholangitis
ROC: receiver operating characteristics
US: ultrasound

## 1 Introduction

Primary sclerosing cholangitis (PSC) is a chronic progressive disease of the intra‐ and/or extrahepatic bile ducts. Sclerosis of bile ducts leads to cholestasis, liver injury, and eventually cirrhosis [1, 2, 3, 4, 5]. The median transplant‐free survival is 10 to 22 years [1, 3, 4, 6, 7]. The main reason for a reduced survival in PSC is the high risk of hepatobiliary malignancy [8, 9, 10]. This includes an increased risk of developing gallbladder carcinoma with a cumulative 10‐year risk of around 2% [11]. Gallbladder carcinomas can develop from gallbladder polyps through a dysplasia‐carcinoma sequence [12]. Gallbladder cancer is an aggressive and difficult to treat malignancy and people with advanced gallbladder carcinoma have a poor prognosis with < 5% 5‐year survival [13]. In the early stages of the disease, cholecystectomy is a curative therapy [2, 13]. Therefore, in people with PSC, surveillance imaging examinations are recommended every 6 to 12 months for early detection of gallbladder polyps [2, 10, 14]. However, data on polyp size indicating a high probability of malignancy are scarce and clinical practice regarding decisions to recommend surgery are highly variable [15, 16]. Additionally, in advanced stages of PSC, cholecystectomy is associated with an increased morbidity [16] and risks have to be weighed against the expected benefits of the procedure.

We here aimed to assess the value of ultrasound and MR imaging for the detection of gallbladder polyps in people with PSC and to define the optimal size cut‐off indicating a high probability of malignancy.

## 2 Patients and Methods

### 2.1 Study Population

**FIGURE 1:**
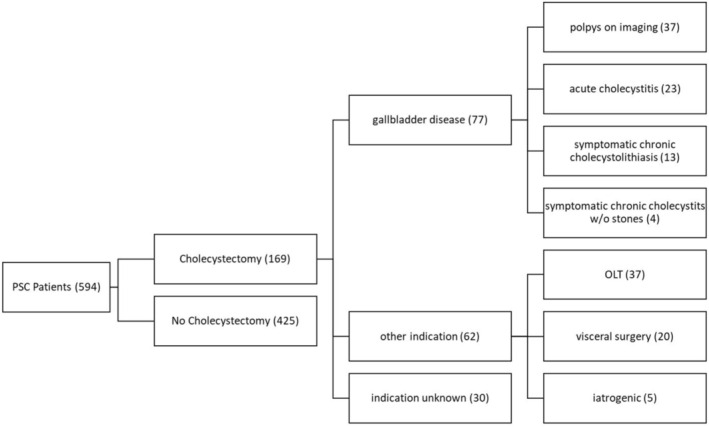
People with PSC treated at the University Medical Center Hamburg‐Eppendorf during the study period (exploration cohort). OLT = orthotopic liver transplantation.

We conducted a retrospective study of people with PSC treated at the YAEL Center for Autoimmune Liver Diseases, University Medical Center Hamburg‐Eppendorf, who received a cholecystectomy between January 2007 and January 2020. Data were retrieved from a registry that includes prospectively acquired data after obtaining written informed consent from patients. The study was approved by the local review board (PV4081). We filtered the registry for patients with the diagnosis “Primary Sclerosing Cholangitis” The medical records were searched for a history of cholecystectomy. To compare the accuracy of ultrasound and MRI for the detection of gallbladder polyps, we included patients with a history of cholecystectomy for polyps and other indications as a control group (Figure 1). In our study, we therefore included patients with a confirmed diagnosis of PSC according to guidelines valid at that time [14], ultrasound and/or MR imaging within 6 months prior to cholecystectomy, and a histology report available from the gallbladder.

Additional data was collected retrospectively from the medical records including imaging modality, presence and size of gallbladder polyps on imaging, and in case of follow‐up visits, the number of follow‐ups as well as size progression of the polyp. Histological results were assessed from pathology reports. Additionally, stage of disease using liver elastography, MELD score at time of cholecystectomy, and history of smoking and alcohol consumption were recorded.

Liver stiffness measurement was performed with FibroScan (EchoSens, Paris, France), as reported previously [17, 18]. The target area of the right liver lobe was 6 cm in depth without major vascular structures and was determined using ultrasound. We included procedures with at least 10 valid measurements, an interquartile range (IQR)/median ratio of less than 30% and a success rate of at least 60%. The median value of liver stiffness was recorded in kPa.

### 2.2 Statistical Analysis

Statistical analysis was performed using IBM Statistics SPSS 27. The level of significance was chosen at *p* < 0.05. We evaluated ultrasound and MR imaging in terms of sensitivity, specificity, and accuracy. The sensitivity of ultrasound and MRI was compared using the McNemar test. Size differences between the 2 groups were assessed using two‐sided T‐test. We grouped the polyps found on imaging into 2 groups based on histology, “malignant findings” and “benign findings”. The “benign findings” group contained all benign findings, including low‐grade dysplasias. The “malignant findings” group consisted of high‐grade dysplasias and gallbladder carcinomas. The optimal size cut‐off differentiating benign from malignant polyps was determined using Receiver Operating Characteristics (ROC) curve.

### 2.3 Multicentric Validation

To validate the cut‐off based on data from Hamburg, we used a multicentre cohort including 17 people with PSC and a history of cholecystectomy for gallbladder polyps from the University Medical Centers in Huddinge, Linköping, Lund, Uppsala and Gothenburg in Sweden and Helsinki in Finland. We assigned the patients into two groups depending on their histological findings, and performed the analysis as mentioned above.

## 3 Results

### 3.1 Study Population

As depicted in Figure 1, we assessed 594 people with PSC treated in the YAEL Center for Autoimmune Liver Diseases at the University Medical Center Hamburg‐Eppendorf. 169 of these patients had their gallbladder removed: 77 because of an underlying gallbladder disease, and 62 in the context of surgery for other indications. In 30 patients, it was not possible to reliably determine the indication for cholecystectomy retrospectively. We focused on the 37 patients who underwent cholecystectomy for polyps on imaging. To assess the sensitivity and specificity of ultrasound and MR imaging for the detection of gallbladder polyps, we included the 102 patients with a history of cholecystectomy for other reasons as a control group.

Three patients did not meet our inclusion criteria. 34 of the patients (20 male, 14 female) with the indication “polyps on imaging” were included in our study. The baseline clinical characteristics are shown in Table 1. The mean age at cholecystectomy was 48 years and the mean duration of PSC at cholecystectomy was 10 years. PSC was not at an advanced stage at the time of cholecystectomy with a median MELD Score of 6 and a median liver stiffness of 7.6 kPa.

**TABLE 1:** Baseline characteristics of people with PSC from the exploration cohort and the validation cohort at the time of cholecystectomy.

People with PSC treated at Hamburg (*n* = 34)	
Age (years)	48 ± 10
Sex	Male 20, female 14
Lifestyle	
History of smoking	6 (17.5%)
History of harmful alcohol consumption^a^	0
Inflammatory bowel disease	25 (75%)
Duration of PSC (years)	10 ± 9 (*M* = 7.5)
MELD score	7.8 ± 3.5 (*M* = 6)
Fibroscan (kPa)	11.5 ± 15 (*M* = 7.6)

People with PSC from multicenter validation cohort (*n* = 17)	
Age (years)	52 ± 14
Sex	Male 12, female 5
Lifestyle	
History of smoking	0
History of harmful alcohol consumption^a^	1 (6%)
Inflammatory bowel disease	13 (76%)
Duration of primary sclerosing cholangitis (years)	10 ± 8 (*M* = 11)
MELD score	6.64 ± 1.9 (*M* = 6)
Fibroscan (kPa)	12 ± 8.3 (*M* = 7.6)

*Note:* Variables are depicted as: Number, (Percentage), ±standard deviation, *M* = median.

^a^ m: > 24 g/d, w: > 12 g/d.

### 3.2 Gallbladder Polyps on Imaging

All 34 patients received ultrasound and MRI examinations before cholecystectomy. We compared their sensitivity and specificity for the detection of gall bladder polyps. All 18 polyps that were identified on histology had been detected using ultrasound. This included all malignant polyps. In 16 of the 34 patients who received cholecystectomy for polyps on imaging, no polyp was detected on histology. This results in a high rate of false positive findings in this group. Within the control group of 102 patients with cholecystectomy for reasons other than polyps, no polyps were seen on ultrasound nor were identified on postoperative histology. Including all patients, ultrasound had a sensitivity of 100% for the detection of polyps on imaging. The specificity of ultrasound was 86% due to false positives on imaging with no polyp identified on histology. The accuracy of ultrasound was 86%. The sensitivity of MRI was 35% (specificity 97%, accuracy 97%): 3 of the 8 malignant polyps were not detected using MRI. Ultrasound was significantly more sensitive than MRI in detecting gallbladder polyps (*p* < 0.001).

### 3.3 Histological Results

**FIGURE 2:**
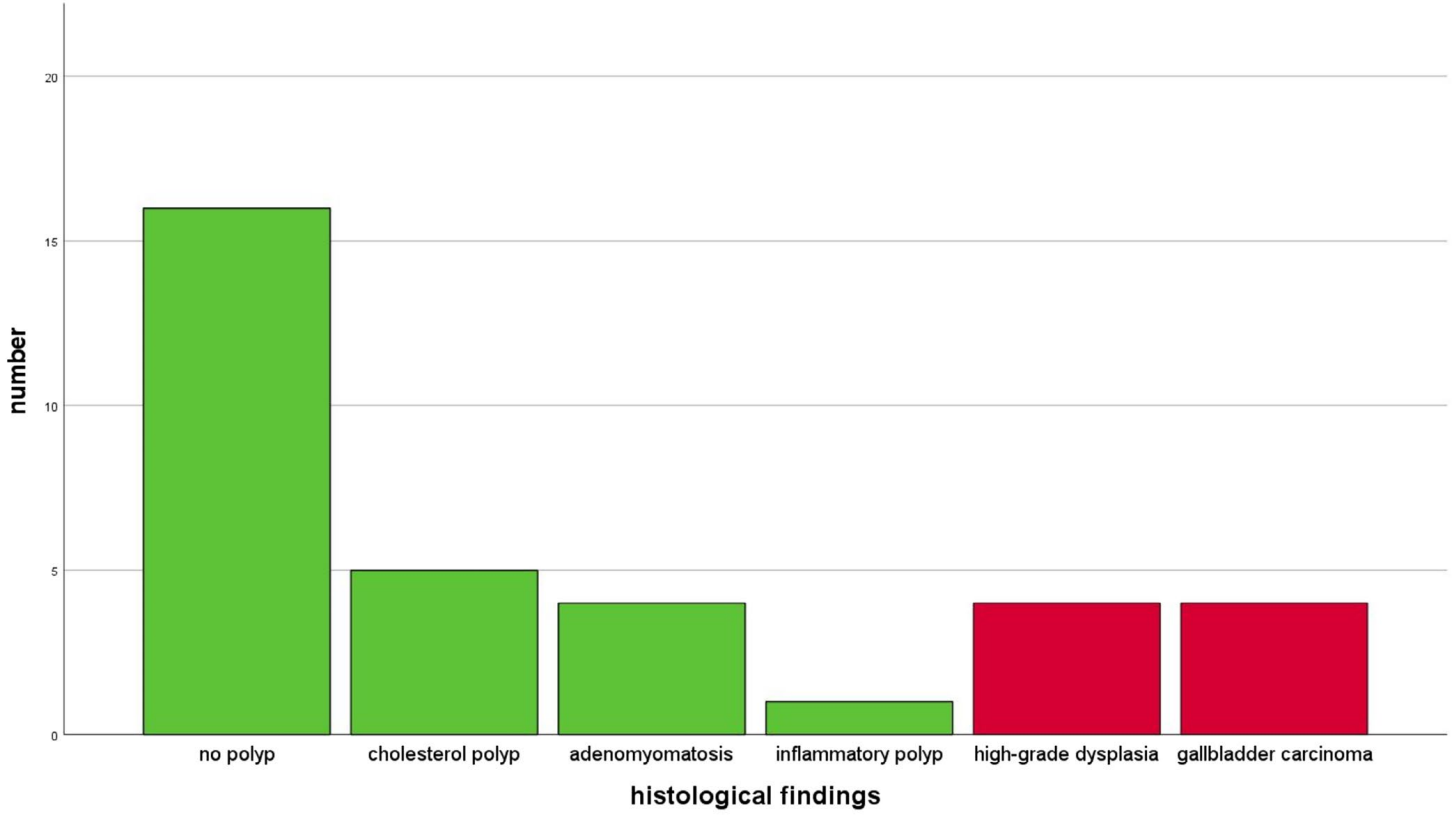
Histological findings after cholecystectomy (exploration cohort).

**FIGURE 3:**
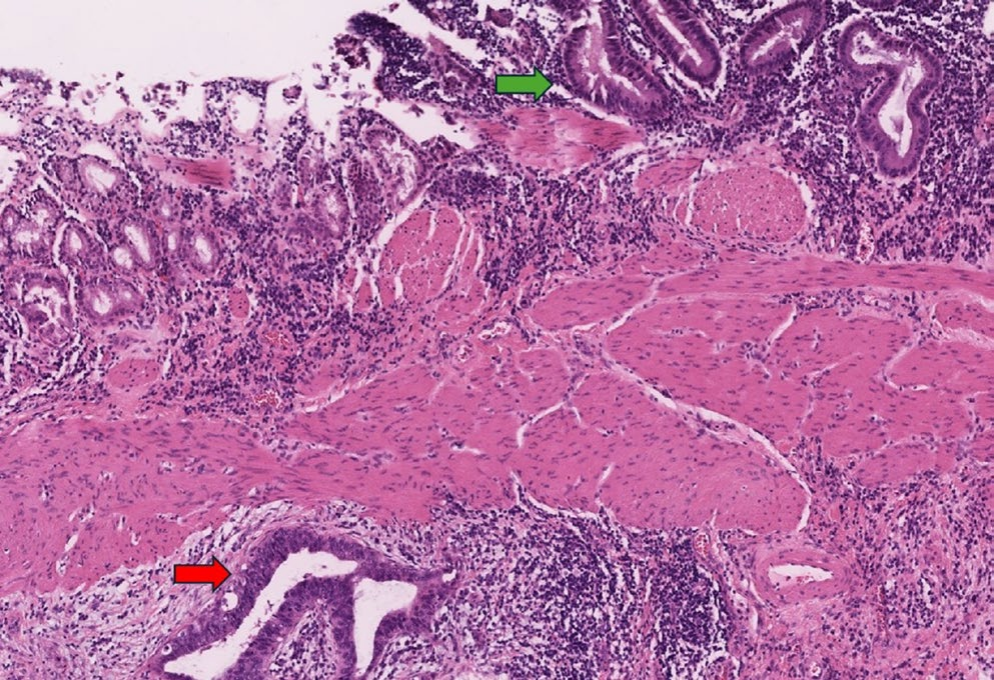
Gallbladder papillary tubular neoplasia with predominantly low‐grade (green arrow) and focal high‐grade dysplasia (red arrow).

The histological results after cholecystectomy are depicted in Figure 2. Eight patients had malignant findings: in four cases, adenoma with high‐grade dysplasia was found, and in the other four, gallbladder carcinoma. Of these, 2 carcinomas each were classified AJCC I and AJCC II. No carcinoma showed lymph node invasion; 1 case showed lymphatic vessel infiltration. All malignant findings were resected R0. Figure 3 shows an exemplary papillary tubular neoplasia with predominantly low grade and focal high‐grade dysplasia.

The benign histological findings included 5 cholesterol polyps, 4 cases of adenomyomatosis, and 1 inflammatory polyp. In 16 patients, no histological correlate to the imaging findings was found. 33 of the 34 patients showed histological signs of chronic cholecystitis.

### 3.4 Correlation Between Polyp Size and Histological Dignity

We divided the polyps into two groups based on histology: malignant and benign findings. We grouped the 4 gallbladder carcinomas and the 4 high‐grade dysplasias into the *malignant findings* group. The *benign findings* group included the polyps with benign histology and the cases where no polyp was found on histology but was seen on imaging beforehand.

**FIGURE 4:**
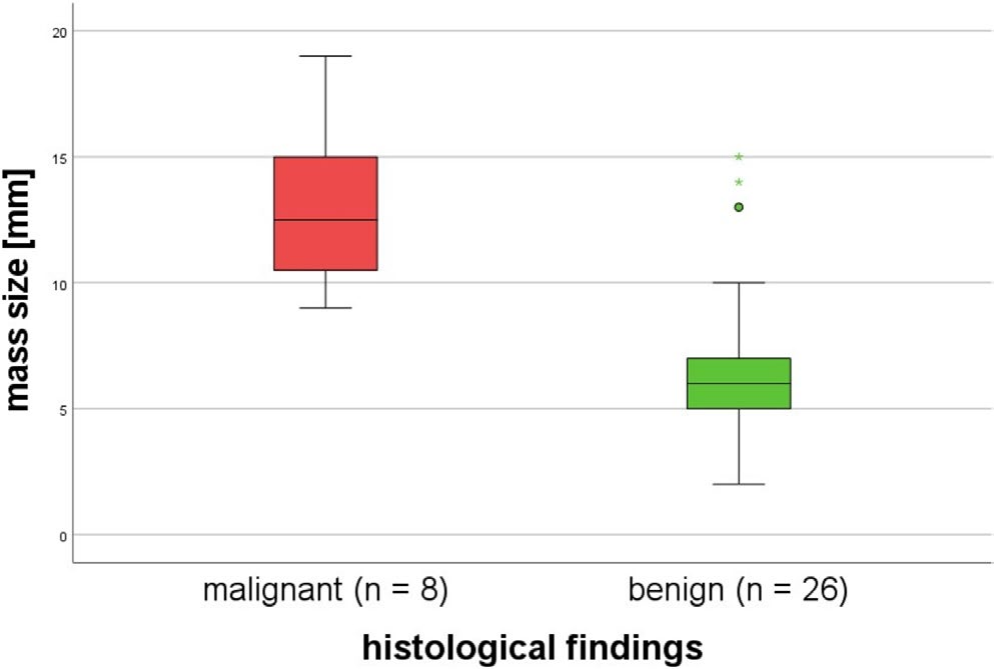
Polyp size as measured by ultrasound for malignant and benign polyps from the exploration cohort.

On ultrasound, the median gallbladder polyp size of the *malignant findings* group (*n* = 8) and the *benign findings* group (*n* = 25), respectively, was 12.5 mm (8.5 mm; 19 mm) and 6 mm (2 mm; 15 mm). Malignant findings were significantly larger than benign findings (*p* < 0.001). Figure 4 compares the polyp size of the two groups. The outliers in the *benign findings* group were two false positive findings with severe chronic cholecystitis and one adenomyomatosis.

The area under the curve (AUC) for the detection of gallbladder malignancy was 0.91 (95% CI 0.82; 1), *p* < 0.001. The best cut‐off value calculated with the Youden Index to differentiate malignant from benign polyps was 8 mm. At this cut‐off value, sensitivity for malignant polyps was 100% and the specificity was 85%. At 8 mm, PPV for a malignant polyp was 66.67% and NPV was 100%.

### 3.5 External Validation Cohort

We next aimed to validate our findings in an external cohort. We included 17 people with PSC who underwent cholecystectomy for gallbladder polyps on imaging at Scandinavian centres. Five patients received both an ultrasound and an MRI within 6 months prior to cholecystectomy, 6 patients received only an ultrasound examination, and 6 patients received an MRI only. In the group that only received an MRI, the polyp size was not reported in 5 cases. Table 1 shows the baseline clinical characteristics of these patients. We assigned patients into 2 groups based on the histological results: the *malignant findings* group and the *benign findings* group. The *malignant findings* group included 4 high‐grade dysplasias and 3 gallbladder carcinomas. The *benign findings* group consisted of 1 adenomyomatosis, 2 inflammatory polyps, 2 hyperplastic polyps, and 5 cases with false positive imaging findings, in whom no polyp was identified on histology.

On ultrasound, the median size of the *malignant findings* group (*n* = 5) was 20 mm (12 mm; 30 mm). The *benign findings* group (*n* = 6) had a median size of 11.5 mm (5 mm; 13 mm, *p* = 0.006).

A difference in polyp size between benign and malignant polyps was also seen on MRI. The median size of the *malignant findings* group (*n* = 4) was 18 mm (10 mm; 70 mm), whereas the *benign findings* group (*n* = 7) had a median polyp size of 6 mm (2 mm; 8 mm, *p* = 0.048).

We calculated the cut‐off value for detecting malignant polyps on ultrasound using a ROC curve. In the multicentric validation cohort, using a cut‐off value of 8 mm, no malignant polyp was missed. Ultrasound was able to distinguish malignant from benign findings with an AUC = 0.92 (95% CI 0.74; 1) and *p* = 0.02.

### 3.6 Growth Progression of Gallbladder Polyps

Looking at the data from all participating centres, 45 patients underwent cholecystectomy after ultrasound showed a gallbladder polyp, 35 patients of these within 6 months of imaging. 10 patients were followed up over at least 12 months prior to cholecystectomy for surveillance and to evaluate growth progression. In two of these 10 patients, gallbladder malignancy was found in histology, and eight cases were benign. We looked at the growth progression of the polyps during the follow‐up of these patients. One case of high‐grade dysplasia was followed up upon for 21 months and showed a growth progression from 6 to 11 mm. One patient developed gallbladder carcinoma during the follow‐up duration of 22 months with a growth progression from 9 to 14 mm. This patient died from liver metastasis of his gallbladder carcinoma. No other patients included in our study developed metastases or died from their gallbladder malignancy. The benign findings showing growth progression during follow‐up included 3 cholesterol polyps, 2 cases of adenomyomatosis, 1 hyperplastic polyp and 2 false positive imaging findings (Table 2). There was no significant difference between benign and malignant findings with regard to the presence of size progression.

**TABLE 2:** Polyp size progression during ultrasound follow‐ups ≥ 12 months.

Histological result	Follow‐up duration (months)	Size progression on ultrasound
High grade dysplasia	21	5 mm (6 mm → 11 mm)
Gallbladder carcinoma	22	6 mm (9 mm → 14 mm)
Cholesterol polyp	12	2 mm (5 mm → 7 mm)
Cholesterol polyp	13	1 mm (5 mm → 6 mm)
Cholesterol polyp	15	3 mm (3 mm → 6 mm)
Adenomyomatosis	23	3 mm (4 mm → 7 mm)
Adenomyomatosis	38	12 mm (3 mm → 15 mm)
Hyperplastic polyp	43	6.4 mm (6.6 mm → 13 mm)
Chronic inflammation	47	7 mm (3 mm → 10 mm)
Chronic inflammation	28	3 mm (3 mm → 6 mm)

*Note:* Size progression is depicted as growth (first ultrasound, last ultrasound before cholecystectomy).

## 4 Discussion

People with PSC are at increased risk to develop gallbladder malignancy. In our study, the most common indication for cholecystectomy was gallbladder polyps detected on imaging in 48% of all cases. We therefore assessed the optimal size cut‐off that can be used to differentiate between benign and malignant polyps. Our results show that the risk of malignancy is low in polyps smaller than 8 mm in size. At 8 mm, the PPV for a malignant finding was 67%. Cholecystectomy is recommended for polyps 8 mm or larger in the current EASL guideline [8] and AASLD practice guidance [19]. However, given the high rate of malignancy in polyps larger than 8 mm found here and reported before [16], it seems advisable to consider cholecystectomy in people with PSC already at smaller polyp sizes. For surveillance, ultrasound or MRI examinations every 6–12 months are recommended according to current guidelines [8]. In our exploration cohort, all patients had received ultrasound and MRI examinations. We are not aware of studies comparing the two imaging modalities for the detection of gallbladder polyps in PSC. Ultrasound detected all gallbladder polyps that were later identified by histology and did not miss any polyps in those patients who underwent cholecystectomy for other indications, resulting in a sensitivity of 100%. The sensitivity of MRI for detecting gallbladder polyps was significantly lower (35%) than ultrasound. There were 16 false positive results in a total of 34 patients with “polyps on imaging” with a polyp seen on ultrasound that did not have a histological correlate. The high sensitivity of ultrasound therefore comes at the cost of many false positive findings. This finding is supported by similar results of a study performed by van Erp et al., who found that 59% of polyps on imaging had no histological correlate [20]. To improve the accuracy, contrast enhanced ultrasound (CEUS) could be used in addition to native ultrasound. Measurement of vascular stalk width using CEUS showed to be superior to grayscale ultrasound in distinguishing adenomatous polyps from cholesterol polyps [21] and may be useful to identify patients in whom intervention is indicated and beneficial. This is also supported by our own more recent clinical experience. In addition, van Erp et al. mention morphology on imaging as a possible indication of malignancy. Interval growth could not reliably distinguish between benign and malignant polyps in their study but in our opinion should raise suspicion of malignancy and close monitoring if cholecystectomy is not performed [20].

In clinical practice, it is of utmost importance to detect polyps that harbour malignancy. Using MRI, three out of the eight malignant polyps were not detected. Thus, screening by MRI alone may not be suitable for the surveillance and early detection of gallbladder malignancies. We did not perform quality assessments of the examinations performed and no central reading of images, as this represents a real‐world study.

Available data indicate that the two imaging modalities may have complementary roles for the surveillance of hepatobiliary malignancy in PSC: ultrasound being more sensitive for the detection of gallbladder polyps, and MRI more sensitive for the detection of cholangiocarcinoma, as recently reported [22]. However, yearly MRI surveillance in people with PSC over a period of 5 years did not result in higher rates of curative therapies for hepatobiliary malignancy in a recent prospective Swedish study [23].

Cholecystectomy in PSC has to be weighed against the expected benefit, especially in advanced stages of disease (Child Pugh B or higher) where it is often associated with increased morbidity [16]. Additionally, the gallbladder may have a protective function in people with PSC. In a recent study, patients with an intact gallbladder had less severe bile duct strictures and dilations compared to cholecystectomized patients [24]. Therefore, prediction of the dignity of a polyp detected on imaging is paramount to guide patient counselling prior to potential surgery. An easily determined parameter for differentiating between malignant and benign findings is polyp size. Our data show that histologically proven malignant polyps were significantly larger than benign polyps. With an AUC of 0.91 (95% CI 0.82, 1; *p* < 0.001), ultrasound seems highly suitable for predicting malignant polyps and the best cut‐off size for the prediction of malignancy was 8 mm. Using this cut‐off, all malignant polyps were detected with a sensitivity of 100% and specificity of 85% and the PPV for a malignant polyp was 67%. We were able to confirm this cut‐off in our independent validation cohort including 17 patients from Scandinavian centres. Although overall polyp size was reported larger in this cohort than for the Hamburg cohort, with an AUC of 0.92 (95% CI 0.74; 1, *p* = 0.02), the validation cohort confirmed that polyp size can distinguish well between malignant and benign polyps with no malignant polyp presenting smaller than 8 mm in the combined cohorts. Our results confirm the single centre data from Eaton et al., who also reported an increased risk of gallbladder malignancy in people with PSC starting at a polyp size of 8 mm [16]. It is important to note that this is different to the general population without PSC, where an increased risk of malignancy is assumed for polyp sizes of 10 mm or more [25].

We also examined polyp growth patterns over time. A total of 10 patients from all participating centres were followed up for at least 12 months before undergoing surgery. Both malignant and benign polyps increased in size over time. Both polyps that turned out to be malignant and that were followed for 21 and 22 months before surgery showed an increase in size beyond the cut‐off size of 8 mm. In contrast, 5 of the 8 benign polyps showed no size progression beyond 8 mm. Size progression alone does not seem to be a reliable indicator of malignancy, but in combination with absolute polyp size may provide further evidence of an emerging malignant process. From single case experience, however, we want to caution against withholding cholecystectomy in people with PSC and documented gallbladder polyp growth even below the cutoff of 8 mm [26]. We agree with Eaton et al. who suggested that polyps smaller than 8 mm and without risk signs such as growth progression do not need immediate surgery [16]. In clinical practice, however, the goal is to act before a cancer has occurred. Therefore, we suggest considering cholecystectomy already at polyp sizes below 8 mm, if their presence has been confirmed by CEUS. Polyps smaller than 5 mm and no increase in size over time may be safely followed up. A prerequisite for this approach is close sonographic monitoring of those gallbladder polyps. For this, a yearly interval between examinations may not be sufficient due to the existing risk of a rapidly growing lesion. We believe that in this setting, ultrasound examinations at least every 6 months should be recommended [26].

Overall, our results show that ultrasound is a reliable method to detect gallbladder polyps and to determine their risk of malignancy. Of a total of 15 malignant polyps, only 1 patient died of his gallbladder carcinoma. For the 14 other patients, early detection and curative surgery resulted in a favourable outcome.

Currently, the guidelines of the British Society of Gastroenterology recommend considering cholecystectomy when a polyp is first diagnosed in people with PSC, regardless of size [1, 2, 8, 14]. It is agreed upon that the risk of underlying malignancy increases with polyp size. The EASL and AASLD guidelines recommend cholecystectomy especially for polyps larger than 8 mm or showing size progression [8, 19]. These recommendations so far were based on poor evidence and derived from a single study [8]. With our data, we validate the existing data and contribute to more informed decision‐making in the treatment of people with PSC.

Our study has strengths and weaknesses: as a strength, we looked at a large group of people with PSC with cholecystectomies and included an independent multicentre validation cohort. As a weakness, we only included patients who had undergone cholecystectomy; patients undergoing follow‐up examinations for polyps on imaging without subsequent surgery were not included due to a lack of histological confirmation of dignity. Also, not all patients in the validation cohort had both ultrasound and MRI before surgery. Additionally, inherent limitations of our study include the retrospective design, the lack of quality assurance and central reading of imaging performed, and the extraction of data from records only.

In conclusion, we here add to the literature on the value of ultrasound for the detection of malignant polyps in people with PSC. Our data indicate that polyps larger than 8 mm have a high risk of malignancy and require cholecystectomy. However, in order to prevent the development of malignancy, we suggest consideringsurgery already for polyps smaller than 8 mm if confirmed on CEUS, and if not performed, close ultrasound follow‐up is recommended. Our data should be further confirmed in independent cohorts and prospective registries.

## Author Contributions

**Johannes Altenmüller:** conception or design of the work, analysis, or interpretation of data for the work, drafting the work. **Christiane Wiegard:** acquisition of data, revising the work critically for important intellectual content. **Marcial Sebode:** acquisition of data, revising the work critically for important intellectual content. **Ansgar W. Lohse:** acquisition of data, revising the work critically for important intellectual content. **Christina Villard:** acquisition of data, revising the work critically for important intellectual content. **Stergios Kechagias:** acquisition of data, revising the work critically for important intellectual content. **Emma Nilsson:** acquisition of data, revising the work critically for important intellectual content. **Fredrik Rorsman:** acquisition of data, revising the work critically for important intellectual content. **Hanns‐Ulrich Marschall:** acquisition of data, revising the work critically for important intellectual content. **Kalle Jokelainen:** acquisition of data, revising the work critically for important intellectual content. **Annika Bergquist:** conception or design of the work, acquisition of data, revising the work critically for important intellectual content. **Martti Färkkilä:** acquisition of data, revising the work critically for important intellectual content. **Christoph Schramm:** conception or design of the work, acquisition of data, drafting the work and revising it critically for important intellectual content.

## Ethics Statement

The study was approved by the local review board (PV4081).

## Consent

Written informed consent from patients was obtained.

## Conflicts of Interest

The authors declare no conflicts of interest.

## Data Availability

The Hamburg cohort data has been presented at EASL 2022.
